# Metacognitive therapy versus cognitive–behavioural therapy in adults with generalised anxiety disorder

**DOI:** 10.1192/bjo.2018.54

**Published:** 2018-09-11

**Authors:** Hans M. Nordahl, Thomas D. Borkovec, Roger Hagen, Leif E. O. Kennair, Odin Hjemdal, Stian Solem, Bjarne Hansen, Svein Haseth, Adrian Wells

**Affiliations:** Professor, Department of Mental Health, Norwegian University of Science and Technology and Research Director, St Olavs Hospital, Nidaros DPS, Norway; Professor, Department of Psychology, Penn State University, USA; Professor, Department of Psychology, Norwegian University of Science and Technology, Norway; Professor, Department of Psychology, Norwegian University of Science and Technology, Norway; Professor, Department of Psychology, Norwegian University of Science and Technology, Norway; Associate Professor, Department of Psychology, Norwegian University of Science and Technology, Norway; Associate Professor, Department of Psychology, University of Bergen, Norway; Clinical Consultant, St. Olavs Hospital, Nidaros DPS, Norway; Professor, School of Psychological Sciences, University of Manchester and Greater Manchester Mental Health NHS Foundation Trust, UK

**Keywords:** Generalised anxiety disorder, metacognitive therapy, cognitive–behavioural therapy, randomised controlled trial, anxiety disorders

## Abstract

**Background:**

Cognitive–behavioural therapy (CBT) is the treatment of choice for generalised anxiety disorder (GAD), yielding significant improvements in approximately 50% of patients. There is significant room for improvement in the outcomes of treatment, especially in recovery.

**Aims:**

We aimed to compare metacognitive therapy (MCT) with the gold standard treatment, CBT, in patients with GAD (clinicaltrials.gov identifier: NCT00426426).

**Method:**

A total of 246 patients with long-term GAD were assessed and 81 were randomised into three conditions: CBT (*n* = 28), MCT (*n* = 32) and a wait-list control (*n* = 21). Assessments were made at pre-treatment, post-treatment and at 2 year follow-up.

**Results:**

Both CBT and MCT were effective treatments, but MCT was more effective (mean difference 9.762, 95% CI 2.679–16.845, *P* = 0.004) and led to significantly higher recovery rates (65% *v*. 38%). These differences were maintained at 2 year follow-up.

**Conclusions:**

MCT seems to produce recovery rates that exceed those of CBT. These results demonstrate that the effects of treatment cannot be attributed to non-specific therapy factors.

**Declaration of interest:**

A.W. wrote the treatment protocol in MCT and several books on CBT and MCT, and receives royalties from these. T.D.B. wrote the protocol in CBT and has published several articles and chapters on CBT and receives royalties from these. All other authors declare no competing interests.

Generalised anxiety disorder (GAD) is a prevalent and disabling disorder characterised by persistent worrying, anxiety symptoms and somatic complaints. An intense experience of uncontrollable and distressing worry over a number of topics is at the centre of the disorder.^1^ GAD impairs work and social functioning with increasing severity if left untreated.^2^ It is one of the most frequent anxiety disorders in primary care, being present in 22% of primary care patients who suffer anxiety and depressive problems.^3^ There are eight meta-analyses of randomised controlled trials of GAD in adults^4^^–^^11^ and two in older adults.^12^^,^^13^ Most published trials have tested the effects of different drugs, different types of cognitive–behavioural therapy (CBT) or treatments using relaxation therapies. Only two meta-analyses have a broader approach and assess all psychological treatments,^7^^,^^11^ and only one includes both CBT and pharmacotherapy, predominantly benzodiazepines.^5^ The psychotherapy of choice for GAD is CBT,^14^ which is the most empirically supported treatment.^10^^,^^11^ CBT produces significant improvement in GAD at 2 years follow-up,^15^ and approximately 50% of patients are clinically improved.^7^^,^^16^ A more recent approach to GAD that appears to be particularly effective is metacognitive therapy (MCT).^17^ Data from randomised trials of anxiety and depression have shown recovery rates in MCT of 72–80%. This treatment has demonstrated greater efficacy in GAD compared with applied relaxation^18^ or treatment based on the intolerance of uncertainty model.^19^ MCT is a good comparator for CBT as MCT appears effective and does not include cognitive restructuring, exposure, applied relaxation or breathing techniques, which are at the core of CBT, thereby minimising overlap. We set out to address the following question: Which is the most effective, MCT or the gold-standard CBT, when we use the same therapists in both conditions under supervision of the originators of these therapies?

## Method

**Fig. 1:**
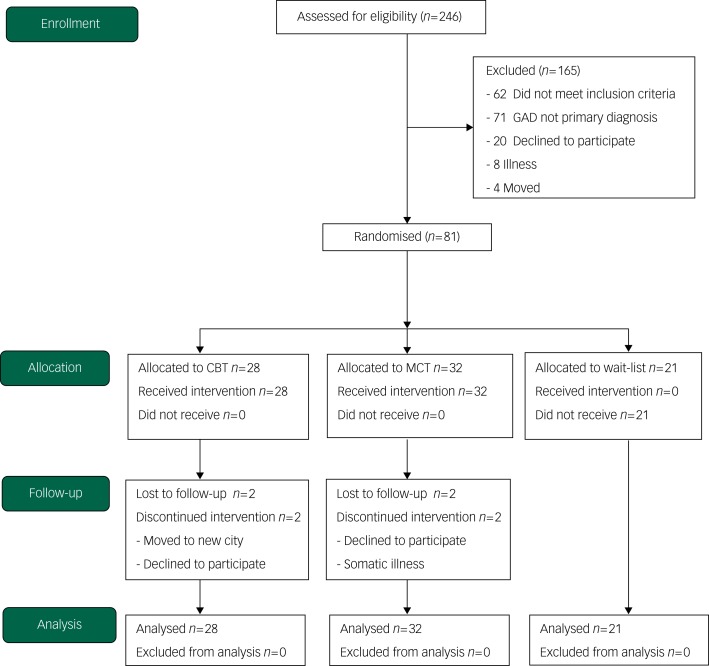
Consort diagram of participant flow (*N* = 246). CBT, cognitive–behavioural therapy; GAD, generalised anxiety disorder; MCT, metacognitive therapy.

The study was conducted at the university outpatient clinic at the Norwegian University of Science and Technology in Trondheim from 2008 to 2016, and was approved by the Regional Committee for Medical and Health Research Ethics (SAK: 4/2006/2369; Clinicaltrials.gov identifier: NCT00426426). A total of 246 patients were referred to the study, and 81 were eligible for, and included in the trial (Fig. 1). They were randomised into three treatment conditions: CBT (*n* = 28), MCT (*n* = 32) and a wait-list control (*n* = 21). Patients were assessed at pre-treatment, post-treatment and at 2 year follow-up. The wait-list participants were offered treatment after 12 weeks post-randomisation. We used a crossover design of therapists to control for therapist factors. Three therapists used CBT and the other three used MCT on the first half of the patients. Halfway into the trial, the therapists were crossed over and delivered the other treatment condition.

The eligibility and inclusion criteria were a diagnosis of GAD, giving written consent before entry in the study and aged 18 years or older. Patients not willing to withdraw psychotropic medication for a period of 3 weeks before entry to the trial were not included but received treatment outside of the trial. The exclusion criteria were known somatic diseases, psychosis, recent suicidal attempts and/or current intent, primary post-traumatic stress disorder, cluster A or cluster B personality disorder, substance dependence or unwillingness to accept random allocation. A summary of the demographic characteristics can be found in Table 1.

**Table 1.** Demographic and clinical characteristics of patients with generalised anxiety disorder in the sample

Characteristic	CBT (*n* = 28)	MCT (*n* = 32)	Wait-list (*n* = 21)	Total (*n* = 81)	F/*χ*^2^	*P*
Age, mean (s.d.)	38.61 (10.9)	36.96 (14.02)	37.86 (12.72)	37.75 (12.54)	0.130	0.878
Female, *n* (%)	19 (68)	24 (75)	16 (76)	59 (73)	0.539	0.764
Social status, *n* (%)					12.31	0.421
Single	3 (21)	7 (50)	4 (29)	14 (18)		
Married/cohabiting	17 (32)	22 (41)	14 (27)	53 (65)		
Separated/widowed	1 (33.3)	2 (66.6)	0 (0)	3 (4)		
Not reported	7 (64)	1 (9)	3 (27)	11 (13)		
Ethnicity, *n* (%)					2.91	0.573
White	27 (96)	31 (96)	20 (95)	78 (96)		
Other	1 (4)	1 (4)	1 (5)	3 (4)		
Work status, *n* (%)					60.80	0.864
Full time/partial	23 (82)	21 (65)	14 (66)	58 (71)		
Unemployed	1	1	1	3 (3.8)		
Student	2	6	4	12 (15)		
Sick leave	2	3	2	7 (8.8)		
Disability pension	0	1	0	2 (2.4)		
Comorbid diagnoses, mean (s.d.)	2.43 (1.26)	2.34 (1.20)	2.52 (1.20)	2.41 (1.21)	0.364	0.696
Major depressive disorder, *n* (%)	14 (33)	15 (35)	13 (32)	42	1.408	0.495
Episode, *n*	2	3	1	6		
Recurrent, *n*	8	11	10	29		
Dysthymia, *n*	4	1	2	7		
Agoraphobia, *n*	2	2	3	7	1.159	0.560
Social phobia, *n*	8	9	7	24	0.188	0.910
Specific phobia, *n*	2	6	1	9	3.194	0.202
Panic disorder, *n*	7	2	3	12	4.166	0.125
Obsessive–compulsive disorder, *n*	1	1	0	2	0.730	0.694
Somatisation/hypochondria, *n*	2	2	2	6	0.203	0.904
Eating disorder, *n*	1	3	1	5	0.966	0.617
Avoidant PD, *n*	2	2	2	6	0.203	0.904
Dependent PD, *n*	2	2	3	7	1.159	0.560

CBT, cognitive–behavioural therapy; MCT, metacognitive therapy; PD, personality disorder.

### Procedures and assessments

The participants were administered a structured interview by independent assessors, and included the Anxiety Disorders Interview Schedule for DSM (ADIS-IV)^20^ and the DSM Structured Clinical Interview for Axis II (SCID-II).^21^ All assessment interviews were videotaped and reviewed to assess diagnostic agreement. Assessors were blind to treatment condition. Independent evaluators were trained and certified on the rating instruments and ADIS-IV and SCID-II assessments. The interrater reliability of independent assessors was based on randomly selected videotaped assessments. The study interrater reliability for a diagnosis of GAD was *κ* = 0.90, and for major depressive disorder it was *κ* = 0.74. After treatment, the evaluators were blinded before assessment.

### Randomisation and masking

Trial participants were randomly assigned to one of three conditions, using the IBM random number generator program. Randomisation was stratified by gender and by DSM-IV^22^ major depressive disorder, and the randomisation sequence was prepared by the trial statistician, who was independent of patient recruitment.

### Therapists

Six clinical psychologists, who had all received extensive training in both CBT and MCT, were selected for the trial. They received regular and equivalent amounts of training and supervision from the originators of the manuals (CBT: T.D.B; MCT: A.W.). All therapists were rated by independent assessors for adherence, competency level and their working alliance with their clients. In addition, by using self-report instruments, the therapists were measured on expectancy of the suitability of the treatment and the credibility in the treatment they were applying to the patient.

### Treatments

We used published treatment manuals of CBT^14^ and MCT.^17^ Treatments were applied for a maximum of 12 weekly sessions of 60 min duration. CBT treatment consisted of four modules: detecting early cues of anxiety and worry, applied relaxation as a response to these cues, imaginal rehearsal of coping methods with self-control desensitisation and CBT on catastrophic beliefs and worry. Patients receiving CBT were informed in the rationale of the treatment that imaginal rehearsal of coping methods would facilitate fear and worry reduction and development of new coping responses, cognitive therapy would reduce anxiety-maintaining thoughts and beliefs and use of cognitive therapy during imaginal rehearsal would provide cognitive coping along with relaxation skills.

We used detection of anxiety cues and applied relaxation in the first three sessions. Self-control desensitisation began in session four, cognitive therapy began in session five and both were conducted in every session thereafter. During desensitisation, after the client was deeply relaxed, external and internal worry cues were presented until the client signalled the presence of anxiety. The client then continued imagining the external situation while imaging that he or she was using relaxation skills in that situation. At the elimination of anxious feelings, he or she imagined continued use of these skills for 20 s and then turned off all imagery and focused only on relaxation for 20 s. The scenes were repeated until the client could no longer generate anxiety or was able to eliminate it rapidly (5–10 s). Cognitive therapy was used up to 15 min per session. The primary goal was to produce cognitive coping responses (both self-statements and perspective shifts) for use during desensitisation; if time or client readiness allowed, we placed emphasis on applying cognitive therapy skills more generally. The cognitive therapy included thought and belief identification, logical analysis with probability, and evidence searching to develop alternative thoughts.

In MCT, the patients' metacognitive beliefs were targeted. Metacognitive beliefs refer to beliefs about thinking, such as the belief that worry is uncontrollable or overthinking is harmful. The metacognitive beliefs were challenged by verbal means and by behavioural experiments with a main emphasis on negative beliefs about worry, specifically its uncontrollability and dangerousness. In MCT the goal is elimination of negative metacognitive beliefs and the introduction of an alternative set of strategies so that the patient is better able to regulate worry and step back from triggering thoughts. MCT for GAD consists of five modules: case formulation and socialisation (sessions one and two), modifying beliefs about uncontrollability and danger of worry (sessions three to six), challenging positive beliefs about the utility and advantages of worry (sessions seven and eight), implementation of alternative coping strategies (sessions nine and ten) and relapse prevention (sessions 11–12). The rationale given to patients is that worrying can be controlled by disengaging from trigger thoughts and postponing further conceptual processing; mental events do not matter, only responses to them do; worrying is harmless and there are no advantages to worrying.

### Measures of outcome

The primary outcome measure was worry severity at post-treatment measured by the Penn State Worry Questionnaire (PSWQ),^23^ a 16-item measure of worry that has evidenced internal consistency, and factor analysis indicated that the PSWQ assesses a unidimensional construct with high convergent and discriminant validity of the measure.^24^ PSWQ was assessed at post-treatment across the three groups and at follow-up for the MCT and CBT groups.

The secondary measures also included: trait anxiety (State-Trait-Anxiety Inventory; STAI-T),^25^ anxiety symptom severity (Beck Anxiety Inventory; BAI)^26^ and interpersonal difficulties (Inventory of Interpersonal Problems; IIP-64).^27^ We also used the Beck Depression Inventory^26^ to control for depressive symptoms.

### Non-specific factors in therapy

Non-specific factors in therapy may vary between the treatment conditions and may be an alternative explanation for any difference in the observed outcome. Thus, we decided to perform a thorough assessment of these potent factors. Therapeutic alliance was measured using the Working Alliance Inventory (WAI).^28^ The WAI was adapted to a rating scale and we used 12 items to rate alliance, with scores from 0–7 on a Likert scale. The patients answered this form at the end of session three.

The therapists' expectancy and credibility in the therapy were measured by The Credibility-Expectancy Questionnaire (CEQ),^29^ which is a self-report measure. Therapeutic expectancy was measured at session three for all therapists and refers to the therapist's predictions about the likelihood that a certain therapy will reduce symptoms. Therapeutic credibility refers to the degree a treatment makes sense to the therapist and seems like a logical and feasible treatment. The CEQ has high internal consistency and high test-rest reliability.^30^ The CEQ is scored on a scale from 0 to 100 of how much the therapist believes in the treatment and in their confidence in being able to implement it.

We measured adherence and competency in CBT with an adapted version of the Cognitive Therapy Competency and Adherence Scale (CTACS),^31^ and in MCT we used the Metacognitive Therapy Competency Scale (MCT-CS).^32^ The independent assessors of the study collaborated with the originators of the treatments in the assessment of the therapists. Each therapist was assessed on 50% of their CBT sessions and 50% of their MCT sessions. Their scores were calculated on both adherence to the protocol and to competency by the checklists for adherence and standardised competency scales. For competency assessed on CTACS in CBT and on MCT-CS in MCT, we used a 0–5 competency level (0, no competency to 5, expert level).

### Statistical analysis

All data were analysed according to the intention-to-treat principle. We had very few missing data and we analysed data with the multiple imputation method for mixed models^33^ and last-observation-carried-forward method. Both methods showed similar results. Categorical data were analysed by chi-squared tests and pre-treatment group differences on unadjusted dimensional self-report measures were tested with analysis of variance (ANOVA). The primary outcome (PSWQ score) and secondary outcomes (STAI-T, BAI and IIP-64 scores) measures were subjected to general mixed-model analysis repeated-measures ANOVA, with treatment condition as the between-groups factor and time (pre-treatment, post-treatment and 2 year follow-up) as the repeated-measures factor. The analyses were conducted separately for the effects at post-treatment and 2 year follow-up (the control group was not measured at follow-up). We also ran analysis of covariance (ANCOVA) on the PSWQ and STAI-T scores, followed with Sidak pairwise *post hoc* comparisons on the adjusted means. Between-group effect sizes are reported as partial eta-squared (*η*_*p*_^2^). We also computed controlled effect sizes with Cohen's formula (*d)*^34^ by subtracting the post-treatment means from the post-wait-list means and dividing this by the pooled s.d. We determined the clinical significance of findings by applying the two-fold Jacobson and Truax criteria (combining criteria a and c)^35^ for defining rates of reliable clinical change and recovery across groups. In a follow-up exploratory analysis we ran a mixed-model ANOVA including the crossover factor (MCT or CBT first/second), to test for any main effects of order of training or interaction between order and therapy type. Because of repeated testing on the primary outcome (PSWQ score), we corrected for multiple comparisons on this measure by Bonferroni correction with a significance level set at *α* = 0.017. The significance level in the secondary outcomes were not adjusted and remained at *α* = 0.05, as these were exploratory and we did not want to force a type II error. We used IBM SPSS version 24 for Windows in the statistical analysis.

### Statistical power

Statistical power analysis was based on the minimum clinically meaningful difference between treatments on the PSWQ, which we set at eight points with a normative s.d. on the PSWQ of 8.^36^ With a probability of type I error of 5% and with the aim of 30 patients included in each of the treatment groups, we calculated the power of the statistical analysis to detect a significant differences to be 0.9314. Thus, with 30 participants in each arm there was a 93.1% chance of detecting a difference at the 5% level of significance between the groups.

## Results

T.D.B. wants to express that he has argued in previous publications that comparative outcome designs should not be conducted because of their potentially limited scientific and applied value. The present study represents an effort, as best as possible, to eliminate the most serious methodological problems commonly associated with those designs. This study is a rare example of having each therapist treat participants in each therapy, thus removing therapist characteristics as a major confounding factor. Also, assessment of therapist competence, as performed in this study, is a move in the direction of reducing the likelihood that differences in quality of the therapies explain any differential outcome. Thus, although other cautions in interpreting the results remain,^37^ the main impediments to unambiguous interpretation of the results have been mitigated.

### Demographic and clinical characteristics

Table 1 provides demographic and clinical data for the sample. There were no significant differences in age (*P* = 0.878), gender (*P* = 0.764), social (*P* = 0.421) or work (*P* = 0.864) status or ethnicity (*P* = 0.573). There were no significant differences in the presence of concurrent major depressive disorder (*P* = 0.495), number of current comorbid disorders (*P* = 0.696), in the distribution of avoidant personality disorder (*P* = 0.904) or in any specific additional disorders among the three conditions.

### Primary outcome

The results of the general mixed-model ANOVA from pre- to post-intervention across the three groups (CBT, MCT and wait-list) showed that there was a significant main effect for group (F(2,78) = 12.573, *P* < 0.01, *η*_*p*_^2^ = 0.244) on the PSWQ (Table 2). There was an overall effect of time indicating significant improvements (F(1,78) = 104.093, *P* < 0.01, *η*_*p*_^2^ = 0.572) and also a significant group×time interaction (F(2,78) = 17.626, *P* < 0.01, *η*_*p*_^2^ = 0.3110).

**Table 2.** Unadjusted means and s.d. at pre-treatment, post-treatment and 2 year follow-up, with pairwise comparisons across assessments

Measure	CBT (*n* = 28), mean (s.d.)	MCT (*n* = 32), mean (s.d.)	Wait-list (*n* = 21), mean (s.d.)	F/*t*	*P*	Pairwise comparisons (*P*)		
						CBT versus MCT	CBT versus wait-list	MCT versus wait-list
Penn State Worry Questionnaire								
Pre-treatment	67.60 (6.28)	65.10 (8.47)	67.52 (7.60)	1.12	0.33	0.461	0.998	0.557
Post-treatment	54.67 (12.80)	42.93 (14.09)	63.95 (8.49)	18.84	<0.001	<0.001	0.034	<0.001
2 year follow-up	53.30 (13.25)	45.41 (15.19)		2.50	0.015	0.015		
Spielberger Trait Anxiety Inventory								
Pre-treatment	57.07 (8.50)	55.76 (9.01)	58.52 (8.06)	0.66	0.519	0.913	0.915	0.588
Post-treatment	47.39 (11.28)	41.78 (12.05)	55.66 (7.19)	10.61	<0.001	0.134	0.027	<0.001
2 year follow-up	45.96 (11.13)	43.53 (9.7)		2.08	0.041	0.041		
Beck Anxiety Inventory								
Pre-treatment	21.46 (10.05)	24.47 (13.82)	28.85 (12.85)	2.14	0.125	0.727	0.121	0.508
Post-treatment	10.42 (12.80)	5.06 (5.57)	22.52 (13.84)	16.53	<0.001	0.170	0.001	<0.001
2 year follow-up	9.85 (9.72)	6.34 (7.82)		1.54	0.127	0.127		
Inventory Interpersonal Problems-64								
Pre-treatment	1.35 (0.55)	1.11 (0.50)	1.31 (0.51)	1.78	0.175	0.225	0.992	0.225
Post-treatment	1.08 (0.54)	0.58 (0.44)	1.19 (0.58)	11.05	<0.001	<0.001	0.815	<0.001
2 year follow-up	1.14 (0.51)	0.69 (0.51)		3.35	0.001	0.001		

Test of significance – two-tailed. CBT, cognitive–behavioural therapy; MCT, metacognitive therapy.

The interaction term demonstrated that there was a different magnitude of improvement across the groups. Inspection of visual plots showed that both treatment groups improved more than the control group, with the MCT condition showing the greatest improvement.

We then calculated follow-up between-group ANCOVAs, controlling for pre-treatment scores on the respective post-treatment outcome variables and computed pairwise *post hoc* Sidak tests on the adjusted means. For PSWQ score, the covariate effect was significant (F(1,77) = 20.838, *P* < 0.01, *η*_*p*_^2^ = 0.213) and there was a significant effect for group (F(2,77) = 18.762, *P* < 0.01, *η*_*p*_^2^ = 0.328). The pairwise tests showed that the CBT group had a lower post-treatment score than the wait-list group (mean difference 9.337, s.e. 3.201, 95% CI 1.524–17.150, *P* = 0.014). Similarly, the MCT group was lower than the control group (mean difference 19.099, s.e. 3.142, 95% CI 11.430–26.768, *P* < 0.01), and crucially, it was also lower than the CBT group (mean difference 9.762, s.e. 2.902, 95% CI 2.679–16.845, *P* < 0.01).

### Secondary outcomes

Next, we ran ANCOVAs on the 2 year follow-up data from the two treated groups only (Table 2). For the PSWQ the covariate effect was significant (F(1,57) = 14.162, *P* < 0.01, *η*_*p*_^2^ = 0.199), as was the effect of group (F(1,57) = 4.339, *P* = 0.04, *η*_*p*_^2^ = 0.071), in favour of MCT. In summary, the tests on the primary outcome showed a better outcome of MCT over CBT post-treatment and this superiority was present at 2 year follow-up.

There was a group main effect on the STAI-T (F(2,78) = 6.168, *P* < 0.01, *η*_*p*_^2^ = 0.137) and an effect of time (F(1,78) = 65.778, *P* < 0.01, *η*_*p*_^2^ = 0.457). The group×time interaction was also significant (F(2,78) = 8.406, *P* < 0.01, *η*_*p*_^2^ = 0.177). In the ANCOVA, controlling for STAI-T at pre-treatment, there was an effect of the covariate (F(1,77) = 28.456, *P* < 0.01, *η*_*p*_^2^ = 0.270) at post-treatment and an effect of group (F(2,77) = 10.709, *P* < 0.01, *η*_*p*_^2^ = 0.218). Pairwise tests showed that scores were lower after CBT than in the wait-list group (mean difference 7.333, s.e. 2.670, 95% CI 0.817–13.850, *P* = 0.02), and also lower after MCT compared with the wait-list group (mean difference 12.094, s.e. 2.613, 95% CI 5.716–18.473, *P* < 0.001). The difference between CBT and MCT was not significant (mean difference 4.761, s.e. 2.393, 95% CI −1.081 to 10.603, *P* = 0.143).

At 2 year follow-up the STAI-T covariate effect was significant (F(1,57) = 8.561, *P* < 0.01, *η*_*p*_^2^ = 0.131), although the group effect failed to reach significance (F(1,57) = 3.956, *P* = 0.05, *η*_*p*_^2^ = 0.065). In summary, although there were lower scores on the STAI-T after MCT and CBT at post-treatment when compared with the wait-list, differences between MCT and CBT at post-treatment and follow-up were non-significant.

The results of the linear mixed-model, repeated measures for the BAI and the IIP-64 were as follows. On the BAI there was a group (F(2,78) = 8.370, *P* < 0.001, *η*_*p*_^2^ = 0.177) and time effect (F(1,78) = 85.391, *P* = 0.01, *η*_*p*_^2^ = 0.523) and a significant group×time interaction (F(2,78) = 8.513, *P* = 0.01, *η*_*p*_^2^ = 0.179). On the IIP-64 the group effect was significant (F(2,78) = 6.329, *P* < 0.01, *η*_*p*_^2^ = 0.140), as was the time effect (F(1,78) = 43.393, *P* < 0.01, *η*_*p*_^2^ = 0.357) and the interaction term (F(2,78) = 6.820, *P* < 0.01, *η*_*p*_^2^ = 0.149).

We conducted ANCOVAs by controlling for the pre-treatment scores. This showed that on the BAI there was an covariate effect (F(1,77) = 25.023, *P* < 0.01, *η*_*p*_^2^ = 0.245) and a group effect (F(2,77) = 16.692, *P* < 0.01, *η*_*p*_^2^ = 0.302), with MCT performing significantly better at post-treatment than both CBT (mean difference 6.673, s.e. 2.474, 95% CI 0.633–12.712, *P* = 0.02) and wait-list (mean difference 15.553, s.e. 2.697, 95% CI 8.969–22.137, *P* < 0.001). CBT was significantly better at post-treatment than the wait-list (mean difference −8.880, s.e. 2.819, 95% CI 1.999–15.761, *P* < 0.01). At 2 year follow-up, MCT was no longer significantly better than CBT (F(1,57) = 3.673, *P* = 0.06, *η*_*p*_^2^ = 0.061).

On the IIP-64 there was an effect of group (F(2,77) = 10.854, *P* < 0.01, *η*_*p*_^2^ = 0.220) and a covariate effect (F(1,77) = 66.830, *P* < 0.01, *η*_*p*_^2^ = 0.465) at post-treatment, with MCT performing better than both CBT (mean difference −0.335, s.e. 0.101, *P* < 0.01) and wait-list (mean difference −0.480, s.e. 0.109, *P* < 0.01). CBT did not perform better than the wait-list (*P* = 0.47). At 2 year follow-up, MCT performed better than CBT (F(1,57) = 7.694, *P* < 0.01, *η*_*p*_^2^ = 0.119; mean difference 0.312, s.e. 0.113, 95% CI 0.087–0.537, *P* < 0.01) and the covariate was significant (F(1,57) = 29.219, *P* < 0.01, *η*_*p*_^2^ = 0.339).

### Non-specific factors and order effects

The trial used six clinical psychologists and all were trained by the originators of the treatment protocols. The therapists were randomised into delivering either CBT first or MCT first, and all therapists swapped treatment condition by mid-trial. The mean score for the CEQ for MCT was 92% (s.d. 6.86, range 80–100) and for CBT it was 87.4% (s.d. 10.74, range 70–100), and the difference on an independent sample *t*-test was not significant (*t* = 1.633, *P* = 0.109).

In CBT the adherence rate was 82% (s.d. 7.31) and the competency level across all six therapists was 3.60 (s.d. 0.56). In the MCT condition the adherence rating was 76% (s.d. 15.00) and competency level was 3.24 (s.d. 0.74). There were significant differences between CBT and MCT in competency level (*t*(31) = 2.063, *P* < 0.01) in favour of CBT, but not in adherence to the protocols (*P* = 0.26). The mean score for the WAI in CBT was 5.54 (s.d. 0.709) and for the MCT group it was 5.75 (s.d. 0.594), with no significant difference between the groups (*P* = 0.23).

To check for order effects, we analysed the PSWQ data (time points 1–3: pre-treatment, post-treatment and follow-up), using treatment order as a category variable, using a 2 groups × 2(order) × 3(time points 1–3) mixed model. The results showed no main effect of order. There was no interaction of order and time (F(2,112) = 0.293, *P* = 0.74, *η*_*p*_^2^ = 0.0050) and no three-way interaction between group, order and time (F(2,112) = 0.264, *P* = 0.76, *η*_*p*_^2^ = 0.005). Therefore, it did not seem to make any difference to the outcome if the therapist delivered a specific treatment first or second.

### Effect sizes, reliable improvement and recovery rates

**Fig. 2:**
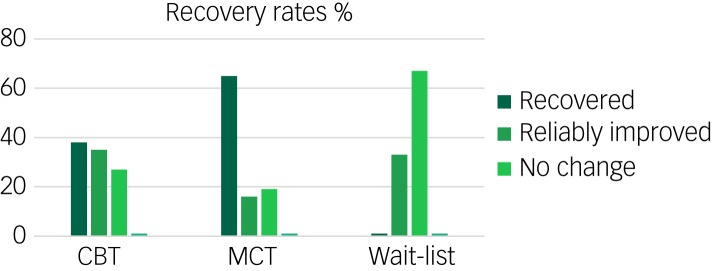
Recovery rates (%) of completers in each condition after treatment measured by STAI-T. CBT, cognitive–behavioural therapy; MCT, metacognitive therapy.

To compare the standardised magnitude of improvement in worry and anxiety symptoms, we calculated pre-treatment to post-treatment and follow-up effect sizes (*d*) with Cohen's formula^33^ on our primary variable, PSWQ score, and we supplemented this with the STAI-T score as this measure is also commonly used in earlier treatment studies, providing a point of reference (Fig. 2). The controlled effect sizes are the most relevant and were calculated as the difference between each active treatment condition at post-treatment and the wait-list expressed in s.d. (pooled) units. For PSWQ at post-treatment, CBT versus wait-list was *d*_*c*_ = 0.86 and MCT versus wait-list was *d*_*c*_ = 1.73. For STAI-T, CBT versus wait-list was *d*_*c*_ = 0.87 and MCT versus wait-list was *d*_*c*_ = 1.42.

To obtain an estimate of the clinical significance of treatment effects on the PSWQ score, we calculated the Reliable Change Index.^34^ We used a twofold criterion here of at least a 2-point s.d. change and also a score below the clinical cut-off. The number of patients meeting these criteria for complete recovery after treatment, according to the PSWQ, was 38% in the CBT group, 65% in the MCT group and none in the wait-list (Table 3). At 2 year follow-up these effects were mainly upheld (31% *v*. 57%). There was a significant difference between MCT and CBT in recovery rates both at post-treatment (*P* < 0.01) and at 2 year follow-up, in favour of MCT (*P* = 0.01).

**Table 3.** Classification of recovery, reliably improved, no change and deteriorated on the Penn State Worry Questionnaire by completers at post-treatment and by 2 year follow-up

	CBT (*n* = 26)	MCT (*n* = 31)	Wait-list (*n* = 21)	*P*
Post-treatment				<0.01
Recovered	10 (38)	20 (65)	0	
Improved	9 (35)	5 (16)	7(33)	
No change	7 (27)	6 (19)	14 (67)	
Deteriorated	0 (0)	0 (0)	0 (0)	
2 year follow-up	CBT (*n* = 26)	MCT (*n* = 30)		<0.01
Recovered	8 (31)	17 (57)		
Improved	10 (38)	10 (33)		
No change	8 (31)	2 (7)		
Deteriorated	0 (0)	1 (3)		

All data shown as *n* (%). CBT, cognitive–behavioural therapy; MCT, metacognitive therapy.

## Discussion

This study compared, for the first time, CBT with MCT in the short- and long-term and controlled for non-specific therapy factors. MCT had better outcomes than CBT on most comparisons. Rates of clinical response, defined as recovery on the PSWQ, demonstrated that overall 65% of patients were recovered after MCT compared with 38% after CBT. The current results for CBT corresponds well with results obtained in other studies and by Borkovec and Costello.^14^ The outcome for MCT are similar to or slightly lower than those obtained in other studies.^18^^,^^19^ This suggests that the outcomes are consistent and representative of the effects normally seen with these treatments.

GAD has high comorbidity with depressive disorder, alcohol, benzodiazepine dependency and somatoform disorders,^1^ and most patients struggle with GAD for 8–10 years, with low rates of remission even when treated. This has led some to characterise it as a chronic disorder.^2^ However, the results of this study combined with other published studies of MCT suggest that CBT and MCT can lead to positive outcomes. Furthermore, these results appear to exist independently of non-specific treatment factors, which appears to support the substantive differential effects of treatment approaches.

Why is there a different response between CBT and MCT? In this study we took great care in training therapists to high standards, using protocols and measuring therapist expectancy and competency as well as assessing the quality of the working alliance. There were few differences in these dimensions across the active treatment conditions. An unexpected difference that did emerge was that therapists were rated as less competent in delivering MCT than CBT. The superiority of MCT in the current trial is therefore unlikely to be because of greater competency levels. It seems more likely that the differences observed can be accounted for by the different focus and techniques used in these therapies. In particular, CBT focuses on reducing the amount of worry through applied relaxation, general cognitive therapy and coping rehearsal during imaginal exposures. In contrast, MCT focuses on reducing worry by modifying metacognitive beliefs about the uncontrollability and dangerousness of worry. CBT focuses predominantly on physiological and emotional cues for anxiety and worry, whereas MCT eschews this aspect and focuses on metacognitive responses such as choosing to leave thoughts alone. Thus, the specific elements in each therapy seem to play an important role and this interpretation becomes more compelling given few observed differences in non-specific factors between conditions, as is the case here. We found that the wait-list condition improved over time. This may reflect spontaneous recovery or regression to the mean, but the results demonstrate the importance of control conditions in interpreting the magnitude of true treatment effects.

Some notable strengths of the study are the use of a randomised controlled design with clearly defined and reliable measures. We used the originators of the treatment modalities in this study to train all therapists up to acceptable standards and assessed allegiance, competence and adherence to the protocol. In a crossover design the therapists swapped treatment modality halfway through the study, so the potential effect of therapist factors was balanced. By balancing the therapist factors, we can reduce the bias in the interpretation of between-condition effects.

There are also some important limitations to consider. First, a significant proportion of the data for 1 year follow-up in the CBT group were not collected at the correct time because of an administrative and logistic failure. Thus, it was decided by the steering group that we should not continue to collect and publish these data, but instead divert all efforts to the 2 year follow-up. Second, the outcome measures were predominantly based on self-report assessments, which could be affected by patient bias. However, Cuijpers *et al*^11^ reported from their recent meta-analysis of GAD that self-report assessments were lower than those of the clinician-rated outcomes (therapist bias). Thus, it seems that patients may underestimate any changes, which indicates that self-reported outcome does not present an inflated result compared with clinician-rated outcome. Third, the level of competency in the methods was unequal, where the level of CBT was rated higher than MCT. This was not in favour of MCT, and therefore greater competency does not seem to explain the superiority of MCT. The difference in competency could be a result of characteristics of the method, the newer and unfamiliar status of MCT or more prior training in CBT.

In conclusion, both CBT and MCT were effective in reducing worry and anxiety symptoms in GAD both in the short- and long-term. However, MCT had a better outcome in reducing worry at post-treatment and in recovery rates at post-treatment and follow-up. In future, highly controlled studies are important to inform healthcare policy on the most appropriate forms of therapy in treating GAD and anxiety disorder in general. In contrast with previous assumptions, GAD can be effectively treated with long-term benefits, and treatments with differential levels of efficacy can be determined. An important implication of these findings is that future meta-analyses will need to take special care in forming meaningful and valid clusters of treatment types for analysis so that important and emerging differences between treatments are not obscured.
